# Supplementary material for: Biochemical Responses in Populus tremula: Defending against Sucking and Leaf-Chewing Insect Herbivores
Source: Plants (Basel). 2024 Apr 30;13(9):1243. doi: 10.3390/plants13091243 (PMC11085190; doi:10.3390/plants13091243)
Supplement: Supplementary file 1 [file plants-13-01243-s001.zip › Supplementary data.pdf]

Table S1 List of compounds monitoring by LC-MS-qTOF in negative ionization mode

	Formula	Theoretical mass	Extracted Mass	m/z	Retention time (min.)	Fragments	Mass error ppm	Score
Catechin	C15H14O6	290.0789	290.0783	289.0718	2.21	289.0715; 245.0819; 203.0716; 151.0402; 125.0244; 109.0296	0.44	97
Chlorogenic acid	C16H18O9	354.0948	354.0941	353.0875	2.23	353.0873; 191.0560; 85.0392	-0.2	98
4-coumaric acid	C9H8O3	164.0474	164.0471	163.9491	2.59	163.0397; 119.0501	0.1	99
Ferulic acid	C10H10O4	194.0579	194.0586	193.0506	2.8	193.0490; 134.0361; 178.0269;	0.15	98
Taxifolin	C15H12O7	304.0583	304.0596		2.90	303.0499; 285.0404; 177.0195; 125.0245	-0.13	99

Rutin	$C_{27}H_{30}O_{16}$	610.1529	610.1607	609.1457	2.93	609.1459; 300.0273	-0.01	99
Procyanidin B1	$C_{30}H_{26}O_{12}$	578.1419	578.1411	577.1347	3.11	577.1343; 451.1019; 425.0876; 289.0713; 161.0243; 125.0242	-0.05	99
Quercetin	$C_{15}H_{10}O_7$	302.0428	302.0420	301.0353	3.75	301.0336; 273.0389; 178.9981; 151.0037; 121.0295	-0.98	99
Kaempferol	$C_{15}H_{10}O_6$	286.0481	286.0476	286.0475	4.13	285.0404; 171.0451; 107.0138	-0.94	99
